# Fruit and vegetables consumption among school-going adolescents: Findings from the baseline survey of an intervention program in a semi-urban area of Dhaka, Bangladesh

**DOI:** 10.1371/journal.pone.0252297

**Published:** 2021-06-08

**Authors:** Marium Salwa, Fatima Subaita, Sohel Reza Choudhury, Md Khalequzzaman, Mohammad Abdullah Al Mamun, Mahfuzur Rahman Bhuiyan, M. Atiqul Haque

**Affiliations:** 1 Department of Public Health and Informatics, Bangabandhu Sheikh Mujib Medical University, Shahbag, Dhaka, Bangladesh; 2 Department of Epidemiology & Research, National Heart Foundation Hospital & Research Institute, Dhaka, Bangladesh; University of Haifa, ISRAEL

## Abstract

**Introduction:**

Interventions aimed at promoting healthy eating habits in adolescence can help prevent chronic diseases and promote healthy ageing. The aim of this paper is to describe the fruit and vegetables consumption habits of adolescents in Dhaka, Bangladesh as well as to identify the socio-environmental, personal, and behavioral factors that influence these habits.

**Materials and methods:**

The baseline data from an intervention study involving 823 grade ten students from two randomly selected secondary schools in a semi-urban area of Dhaka were analyzed. The intake of fruit and vegetables was measured in terms of serving size per day. Hierarchical multiple regression was used to assess the ability of socio-environmental factors such as social support, perceived barriers, and living with patient with chronic diseases; personal factors such as knowledge, self-rated practice, behavioral intention, and body mass index; and behavioral factors such as physical activity, sedentary hours, and sleep duration to predict the level of daily fruit and vegetables intake, after controlling the effect of demographic characteristics of adolescents.

**Results:**

The average daily consumption of fruit and vegetables was 1.22 and 1.99 servings, respectively. Only one-fifth of the respondents (21%) reported eating five servings of fruit and vegetables a day. Inaccessibility at home was reported as the most perceived barrier for both fruit and vegetables intake. Adolescents’ higher fruit and vegetables intake was found to be associated with higher maternal educational attainment, more social support, adequate self-rated practice, positive behavioral intention, higher body mass index, better physical activity, and adequate daily sleeping.

**Conclusion:**

The findings revealed insufficient fruit and vegetables intake among adolescents in a semi-urban area of Bangladesh and associated socio-environmental, personal, and behavioral factors that were utilized in developing an intervention program for this transient age group.

**Trial registration:**

Trial was registered at ClinicalTrials.gov (NCT03975335) https://clinicaltrials.gov/ct2/show/NCT03975335 on June 01, 2019.

## Introduction

A healthy dietary habit is important during adolescence because it has a long-term impact on health and lifestyle. Evidence indicates that dietary habits that lead to non-communicable diseases (NCD) begin in adolescence [1]. Furthermore, dietary habits developed during adolescence have been shown to continue into adulthood [2]. As a result, recent empirical studies emphasize intervening during adolescence to develop a healthy dietary behavior, especially the intake of fruit and vegetables, which is required for preventing NCDs and promoting healthy aging [3].

Unhealthy dietary habits associated with NCD, such as eating less fruit and vegetables, are common among adolescents worldwide [4]. The World Health Organization (WHO) recommends that adolescents consume at least five servings of fruit and vegetables a day [5]. According to the Global School-based Student Health Survey, the majority of adolescents worldwide consume less than the recommended amount of fruit and vegetables, but more carbonated beverage and lipid-rich ready-to-eat processed food [6]. Similarly, the Global Alliance for Improved Nutrition (GAIN) recorded that in Bangladesh, approximately half of school-aged adolescents eat fruit less than once per day [7]. Obesity among Bangladeshi adolescents has been steadily growing in recent years, according to a recent meta-analysis [8], which highlights the critical need to encourage a healthy lifestyle among this transient age group in order to avoid potential NCD.

To design an intervention program for adolescents, a thorough understanding of their dietary behavior is needed. Wilson [9] stresses the importance of culturally tailored interventions based on culture-sensitive theories for successfully modifying dietary behavior in adolescents. Das et al. [10] identified three types of influencing factors when describing adolescent dietary behavior: personal factors such as attitude, belief, self-efficacy, and biological changes; environmental factors such as family, friends, peer networks, school, fast food outlets, and socio-cultural norms; and macro-system factors such as food availability, food production, distribution systems, mass media, and advertising. As a result, planning any intervention program necessitates a comprehensive understanding of the predictors of adolescents’ behavior in the context.

This paper is based on the findings from a base-line survey of a school-based multiple behavior intervention program in Bangladesh aimed at reducing adolescents’ NCD risk behaviors such as unhealthy diet, physical inactivity, and tobacco use. The aim of this paper is to explain adolescents’ fruit and vegetables intake practices and to identify socio-environmental, personal, and behavioral factors associated with adolescents’ fruit and vegetables intake.

## Materials and methods

### Study design and setting

The baseline survey of an interventional study with a before- and-after design is depicted in this article. The research was carried out in a semi-urban area of Dhaka, Bangladesh’s capital. We randomly selected (lottery method) two out of three secondary schools situated in that locality. The detailed methodology of the study is outlined in the protocol paper, which has been published elsewhere [11].

### Participant recruitment and data collection

The sample size for the study was determined expecting a mean difference of 1.48 in the Body Mass Index (BMI) between intervention and non-intervention adolescent groups found in a three months of motivational interview intervention [12]. The sample size was determined using the formula, sample size = 16 ÷ (E/S)^2^ [13], where effect size (E) = 1.48; standard deviation (S) = 5.98; standardized effect size (E/ S) = 0.25; significance level, α (two-sided) = 0.05; β = 1- power = 0.2, non-response rate = 5%. Thus, the calculated sample size was 274 in each group. However, in accordance with the protocol, all 938 students of grade ten were invited to participate in the baseline survey to facilitate the selection of potential recipients for intervention.

Students were invited to participate in the study through academic announcements at least one week prior the date of data collection. Data were collected through a self-administered questionnaire in classroom settings. Students were instructed by the researchers on how to respond to the questionnaire at the outset. Real fruit and vegetables, measuring cups, and pictorial showcards were used to demonstrate the terms used in the questionnaire, such as serving size. School teachers were not allowed in the classroom during data collection to preserve students’ privacy, anonymity, and confidentiality. Students had the provision to ask for any clarification regarding the questionnaire. The students took around 30 minutes to complete the questionnaire.

### Outcome variables

To evaluate adolescents’ consumption of fruit and vegetables, we used the WHO STEPS survey questionnaire [14]. Adolescents were initially asked to record the number of days they eat fruit and vegetables in a typical week, separately. They were then asked to record the amount of serving they consume fruit and vegetables on one of those days. Fruit and vegetables consumption were then measured separately in serving size per day. One serving of fruit was described as one medium-sized piece of fruit (banana, apple, orange, guava, mango, etc.) or a half cup of raw/cooked/canned fruit or a half cup of fruit juice free of artificial flavors. One serving of vegetables was described as one cup of raw, leafy green vegetables (spinach, salad, etc.), or one and a half cup of other cooked or raw vegetables (tomatoes, carrot, pumpkin, beans, gourds, etc. excluding potato), or a half-cup of vegetable juice [14]. Later, the intake of fruit and vegetables in servings per day was summed up to get the total servings a day.

### Demographic factors

The participants’ demographic factors included their sex, parental educational attainment, and occupational status, along with socioeconomic status (SES). We measured SES by assessing wealth index. To construct a wealth index, data on household assets such as table, chair, watch, computer, electricity supply, refrigerator, television, radio, mobile phone, bicycle, and air condition were collected. We used principal components analysis to assign weights to asset variables, as recommended by Filmer and Pritchett [15]. Only the first factor was used to calculate the wealth index, which was then divided into the upper, middle, and lower SES groups.

### Predictor variables

We conceptualized that improved knowledge and attitude would result in healthy behavior change through our intervention program [11]. Hence, we attempted to understand factors that are known to influence dietary behavior like social support, perceived barriers, behavioral intention, and so on. We grouped these variables into three categories, which is described below.

#### Socio-environmental factors

Social support, perceived barriers, and living with any NCD patient were evaluated as socio-environmental factors. The number of sources of social support, such as family members, teachers, health professionals, or peers from whom participants received encouragement to eat fruit and vegetables, was counted. A total of eight sources were identified. Thus, social support was given a score of 0 to 8, with internal consistency reliability coefficient of 0.607 obtained from the Kuder-Richardson or KR-20 test. The commonly perceived barriers to consuming fruit and vegetables were evaluated separately, including inaccessibility at home, high price, tastelessness, unavailability during hunger, and lack of awareness. Students also reported several barriers that were not covered by the questionnaire’s choices. The average number of recorded barriers was used to create a composite variable of perceived barriers. Higher scores indicate more perceived barriers. Adolescents were also asked whether they lived at home with a known NCD patient, and the response was ‘yes’ or ‘no’.

#### Personal factors

Personal factors included knowledge, self-rated practice, behavioral intention, and BMI. A knowledge scale was developed with ten items on the importance of eating fruit and vegetables, and about NCDs, with ‘yes’ or ‘no’ answer options. Correct answers were scored, one for each question, for a total of ten points. The internal consistency reliability coefficient of the knowledge construct was 0.745 in KR-20 test.

Participants were asked to rate their consumption of fruit and vegetables according to their own perception. Thus, the variable self-rated practice was evaluated in two categories- 1. Adequate and 2. Inadequate or uncertain. The behavioral intention towards consuming fruit and vegetables daily in the future was assessed in two categories- 1. positive intention and 2. Negative or no intention. According to the study protocol, BMI was measured using the participant’s weight and height [11].

#### Behavioral factors

Questions assessing physical activity, sedentary hours, and sleep duration were adopted from Global School-based Student Health Survey (GSHS) questionnaire by WHO [16]. In a typical week, physical activity was described as the number of days adolescents recorded walking, running, cycling, playing in the field, swimming, or doing some other form of planned exercise of moderate intensity for at least 60 minutes [16]. Question such as How much time do you usually spend sitting or reclining on a typical day (e.g., watching television, doing computer work, playing video game, chatting with friends, sewing, etc.)?” was used to quantify sedentary hours. Sedentary hours were not described as time spent in the classroom or at home doing homework according to WHO [16]. “How much time do you spend sleeping in a typical day (add night sleep and day nap time)?” was the question used to determine the sleep duration.

The predictor variables are depicted in Table 1.

**Table 1:** Study variables.

Demographic	Socio-environmental	Personal	Behavioral
Sex Father’s education Mother’s education Father’s occupation Mother’s occupation SES	Social support Perceived barriers Living with NCD patient	Knowledge Self-rated intake Behavioral intention BMI	Physical activity Sedentary hours Sleep duration

### Ethical consideration

We obtained ethical clearance to conduct the study from the Institutional Review Board (IRB) of BSMMU (BSMMU/2018/5958). Written permission was obtained from the authorities of both schools and informed written assent was collected from every participant prior to participation. The WHO proposed implied consent procedure was applied to take permission from parents or legal guardians of students [17]. We informed parents or legal guardians of the students about the intervention program over telephone and through notice on school diaries. The presence of the students at the day of data collection was regarded as their guardians’ implied consent.

### Data analysis

The daily fruit and vegetables intake of adolescents have been evaluated across various demographic factors and is presented as a mean with standard deviation (SD). A composite variable of low fruit and vegetables intake was created for the adolescents based on the recommendation of eating five servings of fruit and vegetables per day, and comparisons were made using the chi-square test among different demographic groups.

A hierarchical multiple regression model was constructed to assess the ability of socio-environmental, personal, and behavioral factors to predict the variance in adolescents’ fruit and vegetables consumption. Preliminary analyses were performed to ensure that no assumptions of normality, linearity, multicollinearity, and homoscedasticity were violated. Bivariate correlation among the independent variables did not exceed 0.7. The absence of multicollinearity was determined using a tolerance value of less than 0.10 or a VIF value greater than 10. The normal P-P plot of regression standardized residuals of the dependent variable was found to be acceptable. The scatterplot of standardized residuals verses standardized predicted values showed random scatter, thus the assumption of homoscedasticity was met. Demographic factors were entered at step 1, and socio-environmental, personal, and behavioral factors in step 2.

All statistical analyses were done with SPSS 23, and P-value of 5% was considered significant.

## Results

A total of 823 students participated in this baseline survey with a response rate of about 88%. Around 51 percent of them were female. Their ages ranged from 14 to 18, with a mean (±SD) age of 15.67 (0.84) years. The mean daily consumption of fruit and vegetables was 1.22 and 1.99 servings, respectively. Only one-fifth of the respondents (21%) reported eating at least five servings of fruit and vegetables a day (Table 2).

**Table 2:** Adolescents’ fruit and vegetables intake across different socio-demographic factors.

Variables	Participant	Fruit intake (serving/day)	Vegetable intake (serving/day)	Low fruit and vegetables intake (<5 servings/day) n (%)	P- value^a^
	N (%)	Mean (SD)			
**Total**	823	1.22 (1.17)	1.99 (1.91)	647 (78.60)	
**Sex**					
Male	403 (49.00)	1.25 (1.29)	1.95 (1.87)	318 (78.9)	0.454
Female	420 (51.00)	1.20 (1.29)	2.03 (1.96)	329 (78.3)	
**Father’s education**					
Less than secondary	358 (43.50)	1.12 (1.07)	1.95 (1.86)	292 (81.6)	0.042*
Secondary and above	465 (56.50)	1.31 (1.25)	2.02 (1.96)	355 (76.3)	
**Mother’s education**					
Less than secondary	465 (56.50)	1.10 (1.05)	1.91 (1.80)	381 (81.9)	0.005*
Secondary and above	358 (43.50)	1.38 (1.31)	2.10 (2.05)	266 (74.3)	
**Father’s occupation**					
Service	315 (38.30)	1.27 (1.18)	2.02 (1.94)	248 (78.7)	0.511
Business and others	508 (51.70)	1.19 (1.17)	1.98 (1.43)	399 (78.5)	
**Mother’s occupation**					
Homemaker	723 (87.80)	1.19 (1.12)	1.99 (1.92)	573 (79.3)	0.143
Paid job	100 (12.20)	1.45 (1.50)	2.00 (1.86)	74 (74.0)	
**Socio-economic status**					
Lower	274 (33.30)	1.16 (1.22)	1.96 (1.99)	213 (77.7)	0.800
Middle	279 (33.90)	1.26 (1.18)	1.91 (1.85)	223 (79.9)	
Upper	270 (32.80)	1.26 (1.12)	2.11 (1.91)	211 (78.1)	

^a^ P-value is obtained from the chi-square test for low fruit and vegetable intake across different demographic variables.

* Statistically significant at 5% level.

**Fig 1:**
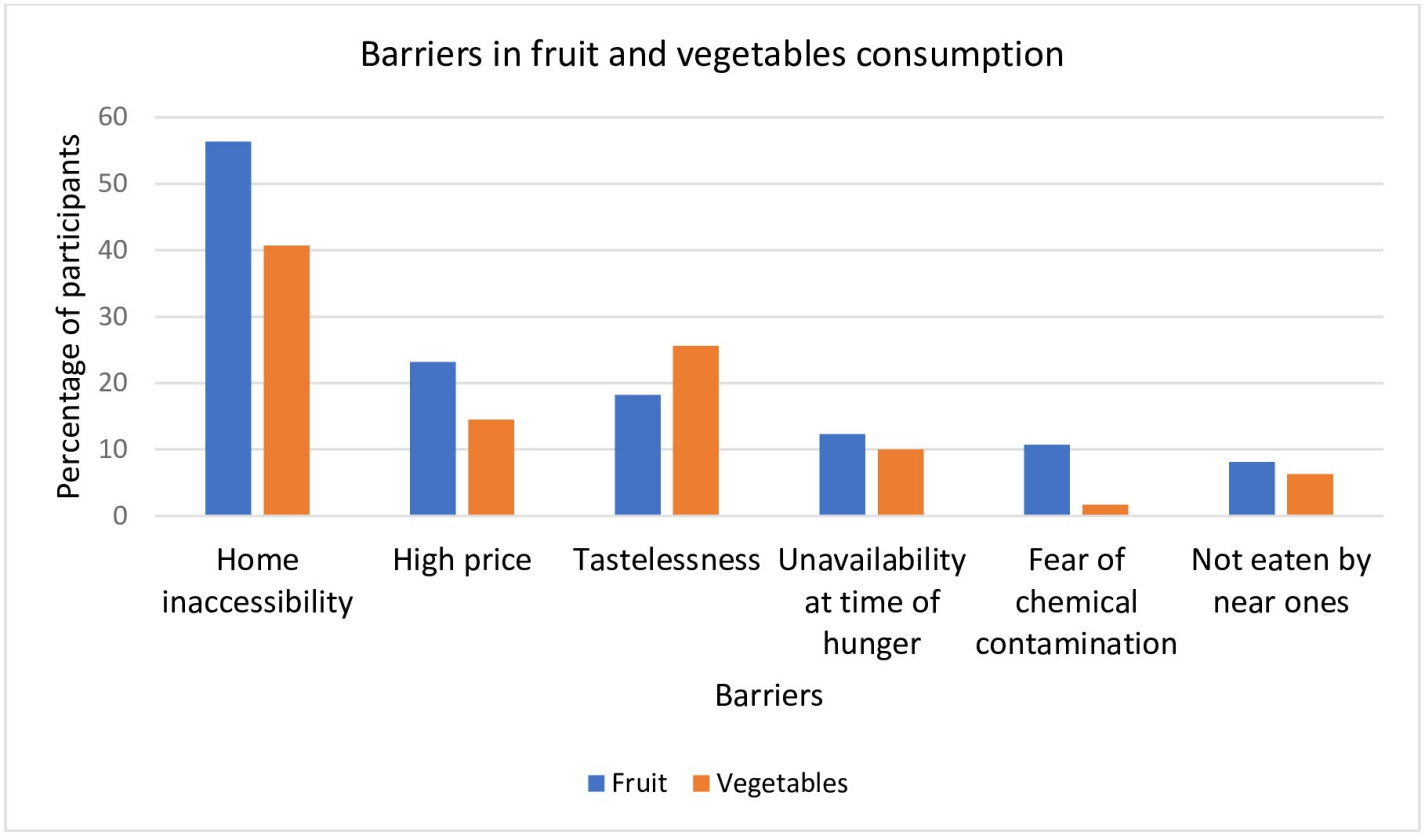
Perceived barriers in fruit and vegetables consumption among participants.

In terms of the perceived barriers to daily consumption of fruit and vegetables, around half of the respondents considered inaccessibility at home as the most important. Other self-reported barriers included high prices, tastelessness, unavailability at times of hunger, and fear of chemical contamination (Fig 1).

Participating students reported an average of about 3 sources of social support they got that encouraged them to eat fruit and vegetables daily, while their average number of perceived barriers was around 2. About 75 percent respondents reported that they lived with at least one patient with NCD at home. Average knowledge score for the participants was 6.72 out of 10. Two fifth of them rated their intake of fruit and vegetables as adequate. However, about 74 percent showed positive intention to consume adequate fruit and vegetables regularly. Mean BMI was 21.27. Students reported an average of 4.7 days of moderate physical activity for at least 60 minutes. Their average sedentary hours and sleep duration were 2.56 and 3.68 hours, respectively (Table 3).

**Table 3:** Statistics of socio-environmental, personal, and behavioral factors.

Variables	Statistics	
	Mean (SD)	Frequency (%)
**Socio-environmental factors**		
Social support	2.96 (1.53)	-
Perceived barriers	2.32 (1.04)	-
Living with NCD patient		
Yes	-	616 (74.8)
No		207 (25.2)
**Personal factors**	-	
Knowledge on importance	6.72 (2.45)	-
Self-rated intake		
Adequate	-	330 (40.1)
Inadequate or uncertain	-	493 (59.9)
Behavioral intention		
Positive intention	-	607 (73.8)
Negative or no intention		216 (26.2)
BMI	21.27 (4.34)	-
**Behavioral factors**		
Physical activity	4.7 (2.78)	-
Sedentary hours	2.56 (1.49)	-
Sleep duration (in hour)	3.68 (1.38)	-

After entering demographic variables at step 1, the model explained only 1% of the variance in fruit and vegetables intake in the hierarchical multiple regression analysis shown in Table 4. After including socio-environmental, personal, and behavioral factors at step 2, the model explained 12.7% of the variance, F (16, 806) = 7.306, and P<0.001. After controlling the demographic variables, added variables at step 2 explained an additional 11.5% of the variance in fruit and vegetables intake.

**Table 4:** Association of socio-environmental, personal, and behavioral factors with fruit and vegetables intake among adolescents.

Variables	Fruit and vegetables intake ^a^ (servings/day)				
	B	SE B	β	95% CI	P-value
**Demographic factors**					
Sex (Female = reference)	0.01	0.17	0.01	-0.33–0.35	0.949
Father’s education (Less than secondary = reference)	-0.06	0.21	-0.01	-0.46–0.34	0.762
Mother’s education (Less than secondary = reference)	0.42	0.20	0.08	0.03–0.81	0.036*
Father’s occupation (Service = reference)	0.02	0.18	0.004	-0.33–0.37	0.900
Mother’s occupation (Homemaker = reference)	0.26	0.26	0.03	-0.24–0.76	0.307
SES (Lower = reference)	0.07	0.10	0.02	-0.13–0.27	0.503
**Socio-environmental factors**					
Social support	0.12	0.06	0.07	0.003–0.24	0.044*
Perceived barriers	-0.08	0.08	-0.04	-0.25–0.08	0.321
Living with NCD patient (No = reference)	0.21	0.19	0.04	-0.17–0.59	0.270
**Personal factors**					
Knowledge on importance	0.04	0.04	0.04	-0.03–0.12	0.267
Self-rated intake (Inadequate or uncertain = reference)	0.92	0.18	0.18	0.58–1.27	0.000*
Behavioral intention (Negative or no intention = reference)	0.87	0.20	0.15	0.49–1.26	0.000*
BMI	0.05	0.02	0.08	0.01–0.08	0.021*
**Behavioral factors**					
Physical activity	0.09	0.03	0.10	0.03–0.15	0.002*
Sedentary hours	-0.06	0.06	-0.04	-0.17–0.05	0.294
Sleep duration	0.12	0.06	0.07	0.001–0.24	0.048*

^a^ R square = 12.7%, Adjusted R square = 10.9%, F (16, 806) = 7.30, p<0.001, R square change = 11.5%, F change (10, 806) = 10.64, p<0.001

*Significant (p<0.05).

Only seven control measures were found to be statistically significant in the final model, with the self-rated intake having the highest beta coefficient value (beta = 0.18, P-value<0.001). Compared to respondents who rated their intake as inadequate or uncertain, we would expect respondents who rated their intake as adequate to consume 0.18 servings more fruit and vegetables a day. Among other personal factors, respondents with positive behavioral intention consumed 0.15 servings more in a day than respondents with negative or no intention. BMI was also found to be significantly associated with higher fruit and vegetables consumption. Every 1 unit increase in BMI resulted in 0.08 serving increase in fruit and vegetables intake. Adolescents’ demographic factors such as mothers’ educational attainment of secondary or above was significantly related to variation in fruit and vegetables consumption. Fruit and vegetables consumption was positively associated with behavioral factors such as regular physical activity and longer duration of sleep. Every 1 day increase in physical activity resulted in 0.1 serving increase and 1 hour increase in sleep duration resulted in 0.07 serving increase in fruit and vegetables intake a day.

## Discussion

This study revealed a low consumption of fruit and vegetables among adolescents in a semi-urban area of Bangladesh. Four out of every five of our respondents reported consuming less than the WHO’s daily recommendation of at least five servings of fruit and vegetables for adolescents [5]. A recent population-based study in Bangladesh [18] reported inadequate fruit and vegetables consumption as the most prevalent adolescent risk behavior, with nine out of ten adolescents consuming insufficient fruit and vegetables. Similar findings have been found in studies conducted around the world [6]. However, we found no difference in low fruit and vegetables consumption between male and female participants of this study.

Rasmussen et al. [19] revealed that age, gender, SES, parental education and occupation, parental consumption, preferences, and home availability- all contribute to adolescents’ eating less fruit and vegetables. Viner et al. [20] also emphasized the importance of various social determinants in shaping adolescents’ healthy dietary behavior. Adolescents with higher maternal educational attainment tend to consume more fruits and vegetables, according to our findings. In a study of Iranian adolescents, Omidvar et al. [21] found that parental education level and occupation significantly influence adolescents’ fruit and vegetables intake. In contrast to these findings, Leroy et al. [22] demonstrated that women’s education or occupation play no role in predicting adolescents’ dietary behavior in rural Bangladesh.

One of the most widely discussed social determinants of adolescents’ dietary intake is SES [20]. However, our study finds no relation between SES and adolescents’ fruit and vegetables intake. This finding is supported by a systematic review conducted by Pearson, Biddle and Gorely [23], who found that SES is unrelated to adolescents’ fruit and vegetables intake. Furthermore, current recommendations put more emphasis on family practices over the role of economic status in the adoption of healthy behavior by adolescents [24].

We found that eating more fruit and vegetables is associated with receiving more social support from family members, friends, schoolteachers, and health workers. Social support is regarded as essential in building adolescents’ self-efficacy to improve dietary behavior. Besides, parental encouragement is proven positively affect children’s fruit and vegetables consumption [23]. Several studies have shown that consistent encouragement from family, school, and community is needed to enhance adolescents’ healthy eating behavior and should be included in any adolescent-focused intervention program [25].

Majority of our study participants reported ‘inaccessibility at home’ as the most frequently perceived barrier to eating fruit and vegetables daily. High cost, unavailability during times of hunger, tastelessness, and not eating daily by the near ones are some other highly rated barriers to adolescents’ daily intake of fruit and vegetables. Around the globe, lack of accessibility is regarded as the most significant perceived barrier to adolescents’ fruit and vegetables intake [26]. Granner and Evans [27] identified that home availability is a significant predictor of adolescents’ fruit and vegetables intake. However, Dizon, Herforth and Wang [28] reported that, despite the availability of varieties of fruit and vegetables in local markets throughout the year, people in Bangladesh usually consume less. This also explains why SES is not associated with adolescents’ fruit and vegetables intake, as the practice of purchasing and storing these foods at home is uncommon in Bangladesh. Thus, in order to minimize adolescents’ perceived barriers to healthy eating, parents as well as community people, should be involved in the intervention program. Furthermore, Cutler et al. [29] found that parent/caregiver and peer support and norms are associated with adolescents’ healthy eating behaviors. Bruening et al. [30] on the other hand, identified perceived barriers as a mediator in the relationship between self-efficacy beliefs and fruit and vegetables intake. Adolescents’ perceived barriers decrease as their self-efficacy beliefs rise and as a result, their consumption of fruit and vegetables increase. Hence, intervention programs aimed at improving adolescents’ dietary behavior should be tailored to boost their self-efficacy in consuming a healthy diet in order to overcome perceived barriers.

Participants of this study who rated their fruit and vegetables intake as adequate reported substantially more consumption than those who rated their intake as inadequate or uncertain. The self-identification of adolescents’ dietary practices affects their dietary habits [31]. Canova [32] explained the relationship between one’s self-rated practice and one’s actual behavior by stating that self-identity as a healthy eater is more likely to develop the intention to consume healthfully, and intention has been shown to bring positive changes in behavior according to the theory of planned behavior [33]. We also found that adolescents with positive behavioral intention consume more fruit and vegetables each day compared to the adolescents with negative or no intention. Thus, self-identified practice influences behavioral intention and indirectly influences adolescents’ dietary behavior [34].

We found physical activity and adequate sleeping as significant predictors of adolescents’ fruit and vegetables consumption. It is widely acknowledged that the determinants of dietary intake and physical activity are multifactorial and interlinked [35]. Thus, the association of adolescents’ fruit and vegetables intake and physical activity has been reported in several studies from various countries [24,36]. Furthermore, the National Sleep Foundation of the United States recommends that teenagers should sleep 8–10 hours a day to promote their health and well-being [37]. Sleep deprivation has been linked to a variety of negative health outcomes, including obesity in adolescents [38]. According to Bel et al., [39] short sleep duration is associated with a poor dietary quality among European adolescents.

The hierarchical regression model used in this analysis revealed that socio-environmental, personal, and behavioral factors predicted more of the participants’ fruit and vegetables intake than their demographic characteristics, which justified our intervention on these factors.

### Limitations

The study findings are limited for several reasons. First, only grade-ten students from two secondary schools in a semi-urban area of Bangladesh took part in this study. Therefore, the results should not be projected to the entire adolescent population of the country. Second, we used self-reported measures to record participants’ fruit and vegetables intake. While we gave continuous instructions on how to measure serving size and helped them recall and calculate their intake over the last seven days, there was still room for certain bias. Third, the hierarchical regression model predicts only 13 percent of the variances in fruit and vegetables consumption by adolescents. However, predicting one’s behavior is complicated. Even with large samples of adolescents, former studies reported less than 10 percent of variances in dietary behaviors, although significant [40].

### Translation into practice

We used the study findings to prepare the content of an intervention program. According to the findings of this baseline study, parents, the home environment, and other social actors play an important role in adolescents’ healthy dietary behavior. However, according to the protocol, we had limited leeway to involve parents and community stakeholders in our intervention program. Hence, we incorporated measures in the intervention to boost participants’ self-efficacy or belief in their ability to always choose a healthy diet for them. The self-efficacy belief is established to minimize one’s perceived barriers to improve healthy behavior. Moreover, we distributed relevant information, education, and communication (IEC) materials in the form of leaflets among participants to take home, expecting that they would indirectly influence their family members. Again, this study found a positive association between behavioral intention and self-rated practice with improved fruit and vegetables intake. As a result, our intervention focused on developing skills in preparing a balanced diet using available foods at home. Furthermore, participants were given practical knowledge about how to choose the right food in right amount for their age and BMI. Hence, their confidence in their self-rated practice as well as their intention to consume adequate fruit and vegetables, are expected to improve. Participants were also given intervention on how to improve their physical activity as well as sleep duration and quality.

## Conclusion

This study revealed insufficient fruit and vegetables consumption among school-going adolescents aged 14 to 18 years in a sub-urban area of Bangladesh. Adolescents’ fruit and vegetables consumption is associated with maternal educational attainment, social support, self-rated practice, behavioral intention, BMI, physical activity, and sleep duration. As a result, some important intervenable predictors of adolescents’ fruit and vegetables consumption is presented in this paper, which can be of much beneficial to design an adolescent-focused intervention program.
